# Root morphological traits and distribution in direct-seeded rice under dense planting with reduced nitrogen

**DOI:** 10.1371/journal.pone.0238362

**Published:** 2020-09-02

**Authors:** Jun Deng, Xiangqian Feng, Danying Wang, Jian Lu, Haotian Chong, Cheng Shang, Ke Liu, Liying Huang, Xiaohai Tian, Yunbo Zhang

**Affiliations:** 1 Hubei Collaborative Innovation Center for Grain Industry, Yangtze University, Jingzhou, China; 2 China National Rice Research Institute, Hangzhou, China; 3 College of Agriculture, Yangtze University, Jingzhou, China; 4 Engineering Research Center of Ecology and Agricultural Use of Wetland, Ministry of Education, Jingzhou, China; University of Tasmania, AUSTRALIA

## Abstract

Water and nutrient absorption from soil by crops mainly depend on the morphological traits and distribution of the crop roots. Dense planting with reduced nitrogen is a sustainable strategy for improving grain yield and nitrogen use efficiency. However, there is little information on the effects of dense planting with reduced nitrogen on direct-seeded inbred rice. Two-year field experiments were conducted with minirhizotron techniques to characterize the root morphological traits and distributions under different nitrogen application rates and sowing densities in two representative inbred rice varieties, Huanghuazhan (HHZ) and Yuenongsimiao (YNSM), grown under three nitrogen application rates (N0: 0 kg ha^–1^, LN: 135 kg ha^-1^, HN: 180 kg ha^–1^) and two sowing densities (LD: 18.75 kg ha^−1^, HD: 22.5 kg ha^−1^). Our study showed that dense planting with low nitrogen improved grain yield partly due to the increased panicle number. The higher sowing density with low nitrogen significantly affected the total root number (TRN), total root length (TRL), total root surface area (TRSA), and total root volume (TRV). There was a significant positive correlation between grain yield and TRL in the 10–20-cm soil layer (*P* < 0.05). The root morphological indexes were positively correlated with dry matter accumulation (*P* < 0.05) and negatively correlated with nitrogen content (*P* < 0.05) at the maturity stage. This study showed that a high sowing density with low nitrogen application can improve root morphology and distribution and increase grain yield and nitrogen use efficiency in direct-seeded inbred rice.

## Introduction

Rice is one of the major cereal crops in the world, and more than 60% of the world’s population consumes rice as their staple food. It is, therefore, of great importance to increase rice yield to ensure food security not only in China but also worldwide [1]. Currently, the main mode of rice planting in China is manual transplanting. This traditional method is highly labor intensive with low productivity; these attributes have influenced the sustainable development of rice production [2].

In recent years, China has witnessed the rapid development of its socioeconomic conditions and a transfer of the rural labor force to urban areas. Direct-seeding technology can reduce labor demands and increase efficiency in rice planting. The advantages of direct-seeded rice are lower labor costs, higher panicle numbers, and a shorter growth duration than in manual and mechanical transplanting methods [3,4]. Therefore, this technology has been popular among farmers, and the area of direct-seeded rice has increased [5].

Nitrogen has a significant effect on root growth and grain yield in rice. When nitrogen is insufficient, the panicle number and number of spikelets per panicle are lower, which can reduce grain yields accordingly [6]. In production, farmers have largely adopted hybrid rice varieties for direct-seeding to obtain higher total dry matter yields with more nitrogen. To realize 10.5–12.0 t ha^-1^ grain yield, 210–260 t ha^-1^ nitrogen is put into use. However, this nitrogen should be utilized properly. Excessive nitrogen can reduce the grain yield and nitrogen use efficiency and even cause environmental pollution [7].

Sowing density has an important effect on direct-seeded rice, and thus, it is an important factor in determining grain yield. Planting density mainly affects the panicle number and the number of spikelets per panicle in direct-seeded rice [8]. At low sowing densities, individual plant growth may be good, but the panicle number is typically low. At high sowing densities, although there are many plants, the excessive population results in more ineffective tillers and a lower grain filling rate, and the occurrence of lodging, diseases, or insect pests can be more serious [9,10]. In production, farmers aim to achieve high grain yields by planting too many seedlings and using excessive fertilizer. As a result, improper nitrogen and density management leads to unhealthy plant populations and frequent pests, diseases and lodging in direct-seeded rice [9].

The root is an important rice organ that performs many physiological functions. Root morphological traits and distribution are closely related to dry matter and grain yield [11,12]. The roots of direct-seeded rice are shallow, and the rice tends to experience lodging. Previous studies have shown that nitrogen application can increase root length and root area in the grain filling stage of rice, which can maintain root activity and delay leaf senescence; as a result, the grain yield can be increased [13]. Root distribution can regulate the magnitude of the leaf angle. A large leaf area index is conducive to ventilation and light transmission, thereby improving photosynthesis after the flowering stage and increasing grain yield. Roots are the main system for absorbing water and nutrients in the later stages of rice growth. Root function is very important in improving the photosynthesis capacity of plants, stabilizing the panicle number, increasing the number of spikelets per panicle, prolonging the function of photosynthesis in leaves, and increasing the grain filling percentage and grain weight [14,15].

Most studies to date have investigated root morphology and physiological characteristics using pot experiments [16,17] and have focused on hybrid rice varieties. In this study, we adopted minirhizotron techniques to conduct field experiments with two inbred rice varieties under different nitrogen and sowing density treatments. The study aimed to 1) compare the grain yield and nitrogen use efficiency of inbred rice under dense planting with reduced nitrogen and 2) characterize the root morphological traits and root distribution of direct-seeded inbred rice.

## Material and methods

### Ethics statements

No specific permissions were required for the activities conducted in this study. The field used in this study was neither privately owned nor protected. The experiments did not involve endangered or protected species.

### Study site

Field experiments were conducted at the experimental farm of Yangtze University (112°31′E, 30°21′N) in Jingzhou City, Hubei Province, China, in 2017 and 2018. The soil in the upper 20 cm at the experimental site was calcareous alluvial with the following properties: pH 6.4, 24.9 g kg^−1^ organic matter, 139.7 mg kg^−1^ alkali-hydrolysable N, 29.5 mg kg^−1^ available P, and 129.8 mg kg^−1^ available K. Soil property data were collected each year and averaged across the two years. The seasonal average maximum and minimum temperatures were 30.7°C and 23.5°C in 2017 and 32.0°C and 24.0°C in 2018, respectively (S1A and S1B Fig). The seasonal average solar radiation was 15.5 MJ m^−2^ d^−1^ in 2017 and 17.2 MJ m^−2^ d^−1^ in 2018 (S1C and S1D Fig).

### Experiments and measurements

Two inbred rice varieties, Huanghuazhan (HHZ) and Yuenongsimiao (YNSM), were used in the experiment. These varieties have similar crop growth durations and are widely planted in Jianghan Plain, China. The experiments were arranged in a split-split plot design with the N treatment as the main plot, planting density as the subplot, and varieties as the sub-subplot. Three replications were performed each year, and the subplot size was 60 m^2^. Two inbred rice varieties were grown at three nitrogen (N) levels, no nitrogen (0 kg ha^−1^ (N0)), low nitrogen (135 kg ha^−1^ (LN)) and high nitrogen (180 kg ha^−1^ (HN)), and two sowing densities (18.75 kg ha^−1^ (LD) and 22.5 kg ha^−1^ (HD)) in 2017. The direct-seeded sowing date was 29 May in 2017 and 2018. The total amount of N was applied in four parts: 50% as a basal treatment, 20% at tillering, 20% at panicle initiation, and 10% as topdressing.

Potassium chloride as well as superphosphate and zinc sulfate were used as the fertilizer sources. For LN: phosphate fertilizer 65 kg ha^−1^, potassium fertilizer base fertilizer 65 kg ha^−1^, and topdressing 30 kg ha^−1^. For HN (at the booting stage): phosphate fertilizer 110 kg ha^−1^, potassium fertilizer 40 kg ha^−1^, and topdressing 50 kg ha^−1^. The ridges were isolated from each other, and the ridges were covered with plastic film.

Two transparent plastic pipes (diameter: 7 cm, length: 1 m) were installed in each plot 2 days after sowing. The depth of the 60-cm root canal was positioned at 30° between the root canal and the ground [18]. The root canal exposed to the ground was coated with an impermeable black adhesive cloth and covered with a cotton ball to prevent light, dust, and water vapor from entering the tube to prevent damage to the instrument and to reduce the influence of external temperatures on the root canal temperature. The roots were scanned with a CI-600 (CID Bio-Science, Camas, WA, USA) root detection instrument system at a scanning angle of 360° at three different stages: 15 days before the heading stage, at the heading stage, and 15 days after the heading stage. The system included a scanner head for collecting images, a laptop computer, and 1-m standard clear soil tubes (50.8 mm internal diameter) with end caps. The root images of the 0–30-cm soil layer were captured with the aid of an automatic indexing handle. The depths of the three soil layers that were assessed were 0–10-cm, 10–20-cm, and 20–30-cm. The area of each picture obtained was 14.1 × 21.6 cm, and the pictures were saved in a BMP file format. The root characteristic data, such as the total root number (TRN), total root length (TRL), total root volume (TRV), and total root surface area (TRSA), were obtained by using WinRhizotrontron ®software (Regent Instruments Inc., Canada image analysis system).

At the heading stage, the plant samples were separated into straw, leaves, and panicles for dry weight determination after oven-drying to constant weight at 70°C. The dried plant samples were ground to a powder using a grinding machine, and the N and carbon contents of the samples were determined using an element analyzer (Costech ECS 4024, Italy).

At maturity, ten hills were sampled diagonally from a 5 m^2^ harvest area to determine the aboveground total dry weight, HI, and yield components. The panicle number was counted for each hill to determine the panicle number per m^2^. The plants were separated into straw and panicles. The straw dry weight was determined after oven-drying at 70°C to a constant weight. The panicles were hand-threshed, and the filled spikelets were separated from the unfilled spikelets by submerging them in tap water. Three subsamples (30 g each) of filled spikelets and three subsamples (3 g each) of unfilled spikelets were used to count the number of spikelets. The dry weights of the rachis and the filled and unfilled spikelets were determined after oven-drying at 70°C to a constant weight. The aboveground total dry weight was the sum of the total dry matter of the straw, rachises, and filled and unfilled spikelets. The number of spikelets per panicle, grain-filling percentage (100 x filled spikelet number/total spikelet number), and HI (100 x filled spikelet weight/aboveground total dry weight) were calculated. The grain yield was determined from a 5 m^2^ area in each plot and adjusted to a standard moisture content of 0.14 g H_2_O.

### Data analysis

Data were analyzed following analysis of Variance, and means of cultivars were compared based on the least significant difference test (LSD) at the 0.05 probability level. The statistical software used for these analyses was SPSS v. 17.0 (IBM Corp., Armonk, NY, USA).

## Results

### Grain yield and yield components

**Fig 1:**
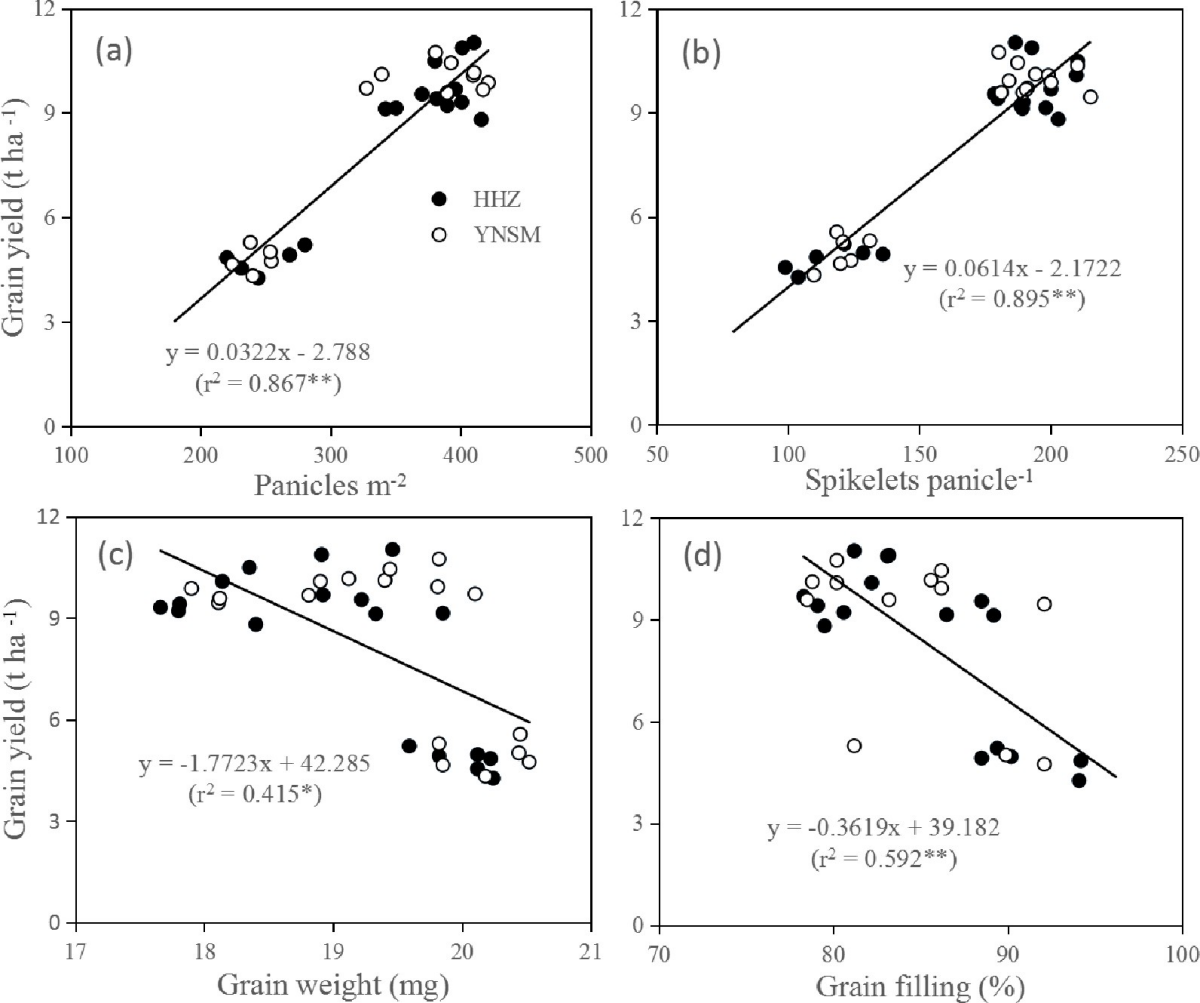
Relationships between grain yield and panicles number (a), spikelets per panicle (b), grain weight (c), and grain filling (d) in HHZ and YNSM.

The N application rate and sowing density significantly (*P* < 0.01) affected the grain yield of inbred rice in the two years (Table 1). HHZ and YNSM both reached the highest grain yield in the LN × HD treatment at 10.14 t ha^-1^ and 10.22 t ha^-1^ in 2017. The grain yield of HHZ showed a positive correlation with the N application rate and sowing density in 2018. The N application rate significantly affected the panicle number in both years, while the spikelet number of HHZ showed no difference in response to the N application rate under the HD treatment in 2018. The panicle number (Fig 1A) and number of spikelets per panicle (Fig 1B) were positively correlated with N application (r^2^ = 0.867, 0.895). Grain weight (Fig 1C) and the grain filling percentage (Fig 1D) were negatively correlated with the N application rate. The panicle number increased significantly with increasing N fertilizer (Table 2), while grain weight showed the opposite trend. The N application rate had no significant effect on the number of spikelets per panicle of either variety under the LN × HD treatment, and there was no significant difference in the grain-filling percentage or grain weight among N application rates.

**Table 1:** Grain yield and yield components of two inbred rice varieties under different nitrogen and sowing density treatments in 2017 and 2018.

Variety	Density	Nitrogen	Panicles m^−2^	Spikelets panicle^−1^	grain filling (%)	Grain weight (mg)	Grain yield (t ha^−1^)
2017							
HHZ	LD	N0	232 ± 12b	104 ± 6b	94.2 ± 0.1a	20.2 ± 0.1a	4.31 ± 0.29b
		LN	354 ± 14a	189 ± 10a	88.1 ± 1.4b	19.5 ± 0.3ab	9.30 ± 0.54a
		HN	398 ± 4a	200 ± 10a	80.3 ± 2.8c	18.2 ± 0.6b	9.59 ± 0.42a
	HD	N0	274 ± 8c	129 ± 7b	89.4 ± 0.9a	19.8 ± 0.3a	5.01 ± 0.51c
		LN	397 ± 15b	197 ± 12a	82.5 ± 1.1a	18.9 ± 0.6a	10.14 ± 0.58a
		HN	396 ± 18a	190 ± 12a	79.7 ± 0.8b	18.0 ± 0.3b	8.91 ± 0.31b
YNSM	LD	N0	240 ± 15b	118 ± 7b	90.3 ± 1.8a	20.2 ± 0.3a	4.29 ± 0.22b
		LN	333 ± 8a	190 ± 5a	86.9 ± 0.9b	19.8 ± 0.4a	9.57 ± 0.23a
		HN	400 ± 14a	201 ± 13a	80.7 ± 2.2c	18.4 ± 0.5b	9.41 ± 0.33a
	HD	N0	246 ± 11c	123 ± 7b	90.2 ± 1.8a	20.2 ± 0.4a	4.89 ± 0.29c
		LN	394 ± 15b	189 ± 10a	84 ± 2.7b	19.5 ± 0.4b	10.22 ± 0.33a
		HN	409 ± 17a	191 ± 9a	79.4 ± 1.2b	18.3 ± 0.5c	9.27 ± 0.45b
2018							
HHZ	LD	N0	197 ± 8c	165 ± 6b	92.8 ± 1.5a	18.9 ± 0.2a	5.98 ± 0.58c
		LN	241 ± 29b	170 ± 7b	92.7 ± 1.6a	18.8 ± 1.0a	8.42 ± 0.53b
		HN	358 ± 2a	191 ± 6a	91.0 ± 2.0b	19.1 ± 0.1a	9.25 ± 0.52a
	HD	N0	245 ± 12c	167 ± 7b	92.7 ± 2.0a	19.0 ± 0.5a	6.24 ± 0.24c
		LN	304 ± 12b	174 ± 9b	93.2 ± 0.8a	18.8 ± 1.0a	9.01 ± 0.28b
		HN	368 ± 31a	183 ± 8a	93.2 ± 1.2a	19.4 ± 1.4a	9.85 ± 1.13a

Within a column for each year, means followed by the same letters are not significantly different according to LSD (0.05).

**Table 2:** Analysis of variance (ANOVA) of the F-values for panicle number, spikelet per panicle, grain filling, grain weight, and grain yield.

Source of Variance	Panicles m^−2^	Spikelets panicle^−1^	Grain filling	Grain weight	Grain yield
Nitrogen (N)	15.50**	11.40**	6.90*	4.40*	24.70**
Variety (V)	9.90*	2.40	4.10	20.10**	27.60**
Density (D)	29.60**	30.20**	29.80**	11.20**	32.70**
N*V	119.40	4.30	0.82	0.30	0.17
N*D	15.60	1.80	0.52	0.52	13.27**
V*D	7.80*	9.80*	43.60**	5.30	14.10*
N*V*D	1.89	40.20	55.60	4.00	6.00

**significant at *P*<0.01

*significant at *P*<0.05; ns, non-significant.

### Dynamic root morphological traits

**Fig 2:**
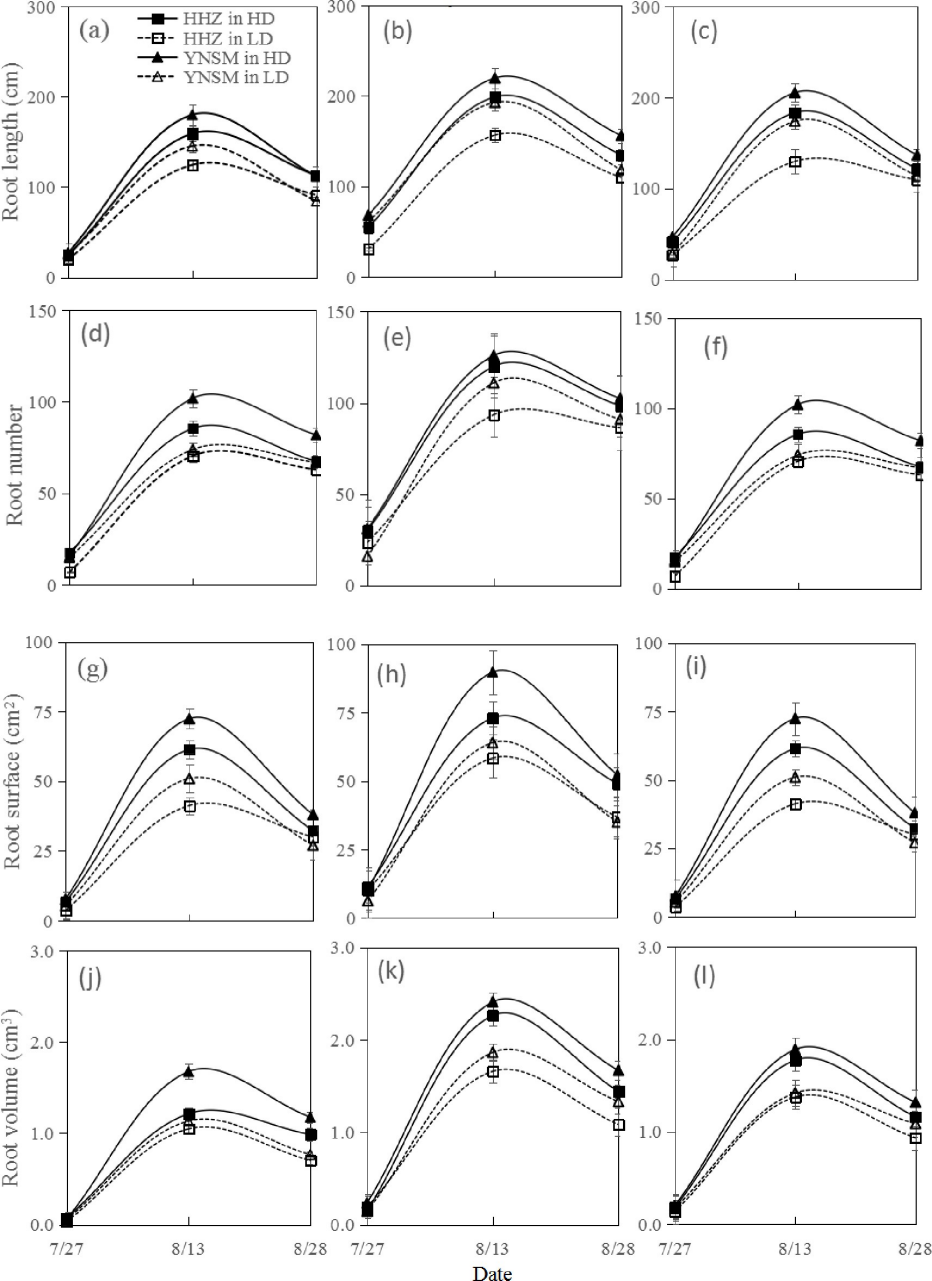
Dynamic root morphological traits of two inbred rice varieties under different nitrogen and sowing density treatments at N0 treatment (a, d, g, j), LN treatment (b, e, h, k), and HN treatment (c, f, i, l).

The N application rate had significant effects on the root morphological traits (Fig 2). The mean TRL of the two varieties increased significantly, by 81.7% and 31.7%, under the LN and HN treatments, respectively, compared with that under N0. N fertilizer had a significant effect on TRN. The mean TRN increased by 68.4% and 27.0% under the LN and HN treatments, respectively, compared with that under the N0 treatment. The mean TRSA increased by 50.1% under the LN treatment and by 28.4% under the HN treatment compared with that under the N0 treatment. The mean TRV of both varieties increased significantly, by 50.6% and 30.8%, under the LN and HN treatments, respectively, compared with that under the N0 treatment. The LN treatment increased the TRL by 58.4% (HHZ) and 105.0% (YNSM), and the HN treatment increased the TRL by 23.0% (HHZ) and 40.3% (YNSM), compared with the N0 treatment.

The sowing density also had significant effects on root morphological traits. Compared with those under the LD treatment, the mean TRL, TRN, TRSA and TRV of both varieties increased significantly, by 27.0%, 33.1%, 40.9% and 25.3%, respectively, under the HD treatment. The interaction between the N application rate and the sowing density significantly affected the root morphological traits. The mean TRL, TRN, TRSA and TRV of the two varieties reached a maximum under the LN × HD treatment.

### Root spatial distribution

**Fig 3:**
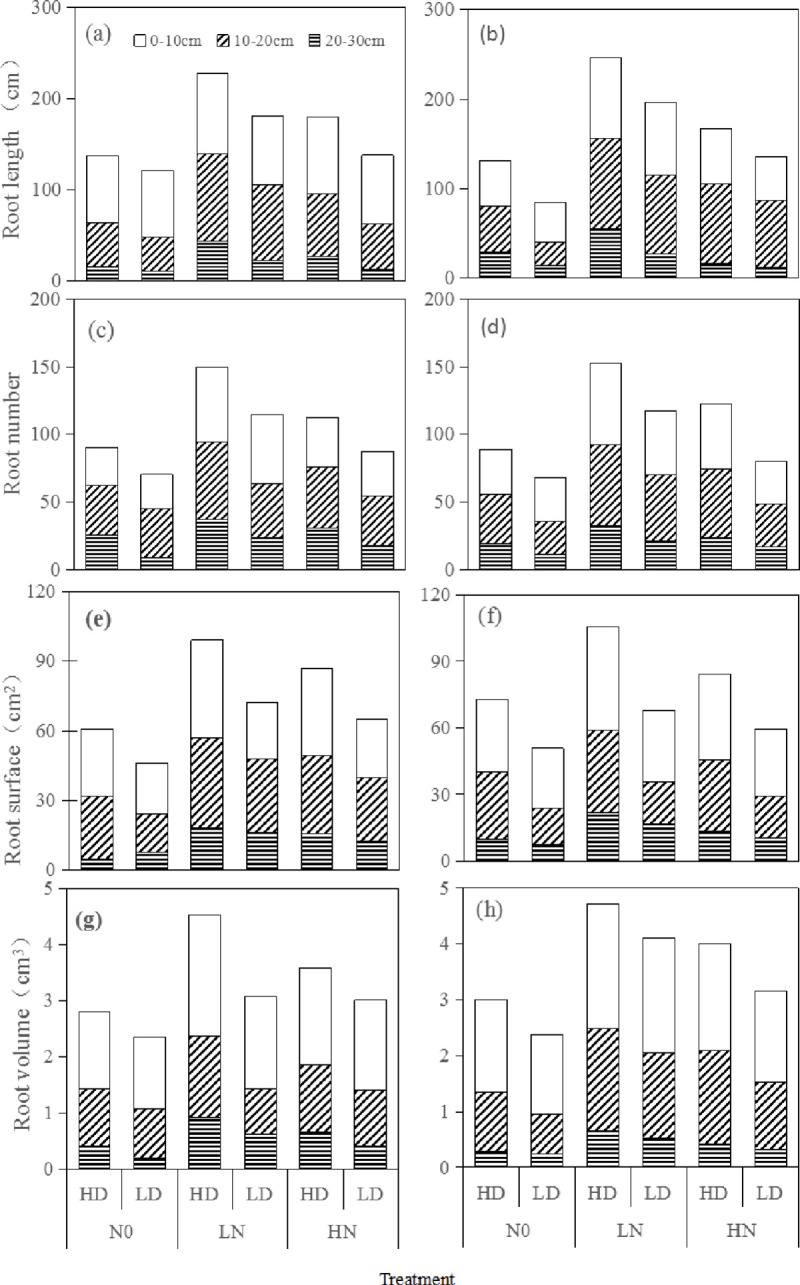
Root spatial distribution (0–30 cm soil layer) of two inbred varieties under N treatments and sowing treatment at flowering stage in HHZ (a, c, e, g) and YNSM (b, d, f, h).

The N application rate had significant effects on the root distribution in different soil layers (Fig 3). The mean TRL, TRN, TRSA and TRV of the two varieties were the highest under the LN treatment and increased significantly across soil layers compared with those under the N0 treatment, especially in the 0–10-cm soil layer. TRL under the LN treatment increased by 111.6% compared with that under the N0 treatment in the 20–30-cm soil layer. TRSA and TRV under the LN treatment increased by 140.8% and 143.3%, respectively, in the 20–30-cm soil layer and by 72.1% and 59.1% under the HN treatment compared with those under the N0 treatment. TRN increased by 80.5% in the 0–10-cm layer under the LN treatment and by 36.2% under the HN treatment compared with that under the N0 treatment.

The sowing density had significant effects on the root distributions in different soil layers. The mean TRL, TRN, TRSA and TRV under the HD treatment were higher than those under the LD treatment. Compared with those under the LD treatment, TRL, TRN, TRSA and TRV increased by 86.0%, 68.5%, 54.4% and 45.4%, respectively, under the HD treatment in the 20–30-cm soil layer.

### Dry matter accumulation and the carbon and nitrogen contents

The N application rate and sowing density had significant effects on dry matter accumulation (*P* < 0.05). Dry matter accumulation increased significantly with the N application rate in both years (Table 3). Sowing density had a significant positive correlation with dry matter accumulation (Table 4).

**Table 3:** Aboveground total dry weight (TDW) at maturity and harvest index of two inbred rice varieties under different nitrogen and sowing density treatments in 2017 and 2018.

Variety	Density	Nitrogen	TDW at heading stage (g m^−2^)	TDW at maturity (g m^−2^)	Harvest index (%)
2017					
HHZ	LD	N0	520 ± 22c	789 ± 244b	51.1 ± 2.7a
		LN	750 ± 22b	1025 ± 27a	50.2 ± 1.6ab
		HN	867 ± 20a	1134 ± 33a	47.9 ± 2.8b
	HD	N0	579 ± 16c	775 ± 34c	50.3 ± 1.9a
		LN	790 ± 45b	1260 ± 28b	49.4 ± 2.3ab
		HN	910 ± 59a	1391 ± 15a	47.1 ± 2.3b
YNSM	LD	N0	580 ± 12c	810 ± 43c	51.0 ± 1.5a
		LN	870 ± 42b	1204 ± 3b	50.1 ± 2.4ab
		HN	931 ± 43a	1144 ± 26a	47.7 ± 2.4b
	HD	N0	591 ± 21c	821 ± 43c	50.5 ± 2.1a
		LN	896 ± 50b	1341 ± 23b	48.2 ± 2.4b
		HN	990 ± 22a	1492 ± 20a	46.2 ± 0.7c
2018					
HHZ	LD	N0	740 ± 26c	798 ± 37c	51.2 ± 2.7a
		LN	1242 ± 30b	1208 ± 49b	50.2 ± 1.3ab
		HN	1342 ± 41a	1469 ± 19a	47.8 ± 1.2b
	HD	N0	730 ± 19c	1068 ± 28c	51.1 ± 2.3a
		LN	1216 ± 54b	1844 ± 65b	49.6 ± 1.2ab
		HN	1300 ± 81a	2045 ± 41a	47.4 ± 2.4b

**Table 4:** Carbon and nitrogen contents in stems and leaves of two inbred rice varieties under different nitrogen and sowing density treatments.

Variety	Density	Nitrogen	Carbon content in stems and leaves (%)	Nitrogen content in stem and leaf (%)
HHZ	LD	N0	35.7 ± 0.8c	0.70 ± 0.01a
		LN	36.7 ± 0.4b	0.60 ± 0.01b
		HN	38.7 ± 0.2a	0.53 ± 0.06c
	HD	N0	35.3 ± 0.5c	0.59 ± 0.01b
		LN	37.0 ± 0.3b	0.70 ± 0.01a
		HN	38.0 ± 0.6a	0.65 ± 0.08ab
YNSM	LD	N0	37.6 ± 0.2a	0.53 ± 0.06c
		LN	37.2 ± 0.5a	0.63 ± 0.05ab
		HN	37.4 ± 0.5a	0.68 ± 0.03a
	HD	N0	36.8 ± 0.6a	0.51 ± 0.04b
		LN	37.4 ± 0.1a	0.54 ± 0.04b
		HN	37.5 ± 0.2a	0.61 ± 0.06a

The N application rate had a significant positive effect on the C content in the stems and leaves of HHZ but had no significant effect on the C content in the stems and leaves of YNSM. The N application rate had a significant effect on the N content in stems and leaves.

### Nitrogen use efficiency

**Fig 4:**
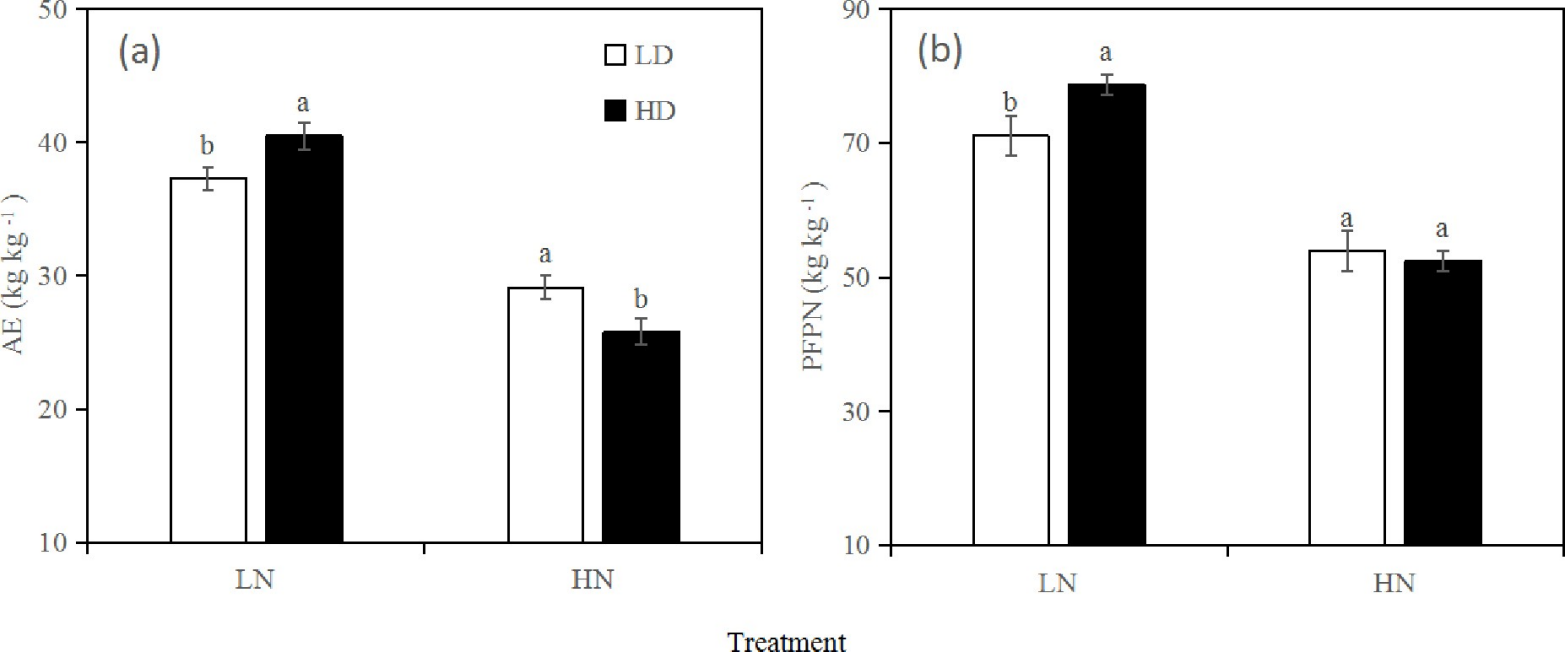
Agronomic efficiency (AE) (a) and partial factor productivity (PFPN) (b) relative to N application rate and sowing density in inbred rice. Within a column, means followed by the same letters are not significantly different according to LSD (0.05).

Significant effects on agronomic use efficiency (AE) and partial factor productivity of nitrogen (PFPN) were observed under different N levels and sowing densities (Fig 4). This study showed that increasing the sowing density had a positive effect on the AE and PFPN of both varieties under the LN treatment but a negative effect on AE and no significant effect on PFPN under the HN treatment.

### Relationship between the various root morphology indexes at different soil layers

TRN, TRL, TRSA, and TRV had significant effects on grain yield (Table 5). The correlation between TRL and grain yield was the highest (r = 0.80) in the 10–20-cm soil layer. At the same time, the root morphology indexes had significant effects on the dry matter accumulation at the mature stage (*P* < 0.05) and were negatively correlated with the N content of the rice (*P* < 0.05). The TRL at 0–10-cm had the highest correlation with dry matter accumulation at the heading stage (r = 0.94). The correlation between the root indexes and dry matter accumulation was the highest in the 10–20-cm soil layer.

**Table 5:** Correlation coefficients (r) between root morphological characteristics and grain yield, dry matter accumulation, and carbon and nitrogen contents.

Root system parameter	Soil layer (cm)	Grain yield	Dry matter at heading stage	Maturing dry matter	Stem and leaf nitrogen content (ns)	Grain nitrogen content
TRL	0–10	0.43 ns	0.47 ns	0.54 ns	0.49	−0.57*
	10–20	0.80**	0.94**	0.91**	0.05	−0.59*
	20–30	0.29ns	0.56*	0.51 ns	0.05	−0.75**
TRN	0–10	0.62*	0.85**	0.80**	0.23	−0.74**
	10–20	0.58*	0.74**	0.76**	0.08	−0.80**
	20–30	0.55 ns	0.64*	0.65*	0.06	−0.63*
TRSA	0–10	0.62*	0.85**	0.80**	0.23	−0.74**
	10–20	0.58*	0.74**	0.76**	0.08	−0.80**
	20–30	0.55 ns	0.64*	0.65*	0.06	−0.63*
TRV	0–10	0.61*	0.83**	0.81**	−0.05	−0.74**
	10–20	0.58*	0.74**	0.74**	−0.23	−0.55 ns
	20–30	0.61*	0.69**	0.71**	0.21	−0.66*

## Discussion

The root morphological and physiological traits were closely related to grain yield [19,20]. A strong root system improves the N absorption capacity of the plant and results in higher grain yields [21,22]. In this study, the TRL, TRN, TRSA, and TRV of the two varieties were found to be closely related to the N application rate. The morphological indexes of roots increase with increasing N application rate [23]. However, excessive application of N did not promote the growth of rice roots or the grain yield. At the same time, the effect of the sowing density on the rice roots was significant, and the morphological index was higher at high sowing density than at low density, which indicated that the increase in sowing density promoted the growth and development of the direct-seeded rice roots.

The effect of rice roots on the growth and grain yield was not only influenced by root morphology but also closely related to root distribution [24]. Previous studies considered that the upper layer (0–20-cm) of the rice roots played an important role in absorbing moisture and nutrients, which can increase the grain filling rate and grain weight [25,26]. Our study showed that different N application rates and sowing densities had significant effects on the root morphology in different soil layers for both varieties. Appropriate increases in nitrogen fertilizer and planting density can improve root morphology. In addition, the correlation between root length and grain yield was the highest in the 10–20-cm soil layer (r = 0.80). The correlations among the TRN, TRSA, and TRV indexes were the highest in the 0–10-cm soil layer. Roots are mainly distributed in the 0–20-cm soil layer, especially the 0–10-cm soil layer, where more than 80% of roots grow [27]. In this study, the 0–10-cm soil layer accounted for 40%–50% of the TRN, while the 10–20-cm soil layer accounted for approximately 30% of the TRN. These findings differed from those of previous studies, which may be attributed to the different varieties used [28].

The TRL, TRN, TRSA, and TRV of both varieties were significantly correlated with dry matter accumulation at the heading stage. The correlation coefficients between root length (10-20-cm soil layer) and dry matter accumulation at the heading and mature stages were R^2^ = 0.94 and R^2^ = 0.91, respectively. The correlation coefficients between dry matter accumulation and TRN, TRSA, and TRV were the highest in the 0–10-cm soil layer. There was a significant negative correlation between root morphology and N content in rice. Inbred rice varieties produce high grain yields due to their high panicle number. Under direct-seeded conditions, the rice roots were shallow. However, the root distribution in the deep soil layer can help to increase the grain yield and nitrogen use efficiency of direct-seeded rice because the deep penetration of rice roots can delay leaf senescence and improve photosynthesis [29]. Therefore, if the root quantity in the deep soil layer of inbred rice can be increased, the TRSA can be expanded and the root nutrition absorption range can be increased, which plays an important role in lodging resistance.

Previous studies have shown that an increase in the N application rate and sowing density can increase the nitrogen transport capacity in leaves, and nitrogen is negatively correlated with PFPN and NUE [30]. Our study shows that increased sowing density can promote dry matter production in direct-seeded rice. N mainly increased the panicle number and number of spikelets per panicle, but the grain filling rate and grain weight decreased. Grain yield is the result of dry matter production, transportation and accumulation. The results showed that an increase in N application (within reasonable levels) could increase the dry matter accumulation of both varieties under direct-seeded conditions. In addition, an increase in the sowing density promoted the dry matter production of the direct-seeded rice, whereby the grain yield also increased. Our study also showed that increasing the N application rate reduced the AE and PFPN and that appropriately increasing the sowing density increased the AE and PFPN.

In addition, the effects of the N application rate and sowing density on the carbon content in the stems and leaves of HHZ were positively correlated, while the N content decreased with increasing N application rate and sowing density. Compared with that under HHZ, the carbon content in the stems and leaves of YNSM did not change significantly with the increase in the N application rate and sowing density, but the N content in the stems and leaves showed significant correlations with the N application rate and sowing density.

In previous studies, researchers investigated roots at different soil layer depths. The correlation between deep root distribution and yield was not found to be consistent. However, the deep root distribution was closely related to the yield [31]. In this study, under the same N fertilizer application rate and sowing density, the root indexes of the 0–10-cm and 10–20-cm soil layers of the two varieties were significantly different, and the root morphology indexes of YNSM were higher than those of HHZ. The nitrogen use efficiency of rice roots is different among different varieties [32]. The root indexes of YNSM were higher than those of HHZ, which may be due to the individual characteristics of the different varieties. Therefore, breeders should focus on deep-rooted varieties to improve grain yield, nitrogen use efficiency and lodging resistance under direct-seeded conditions.

## Supporting information

S1 Fig Daily maximum temperature (●), minimum temperature (○), and solar radiation (▲) in 2017 (a, c) and 2018 (b, d) in Jingzhou, Hubei Province, China. (DOCX)
