# Supplementary material for: Root morphological traits and distribution in direct-seeded rice under dense planting with reduced nitrogen
Source: PLoS One. 2020 Sep 2;15(9):e0238362. doi: 10.1371/journal.pone.0238362 (PMC7467324; doi:10.1371/journal.pone.0238362)

**S1 Fig.** Daily maximum temperature (●), minimum temperature (○), and solar radiation (▲) in 2017 (a, c) and 2018 (b, d) in Jingzhou, Hubei Province, China.


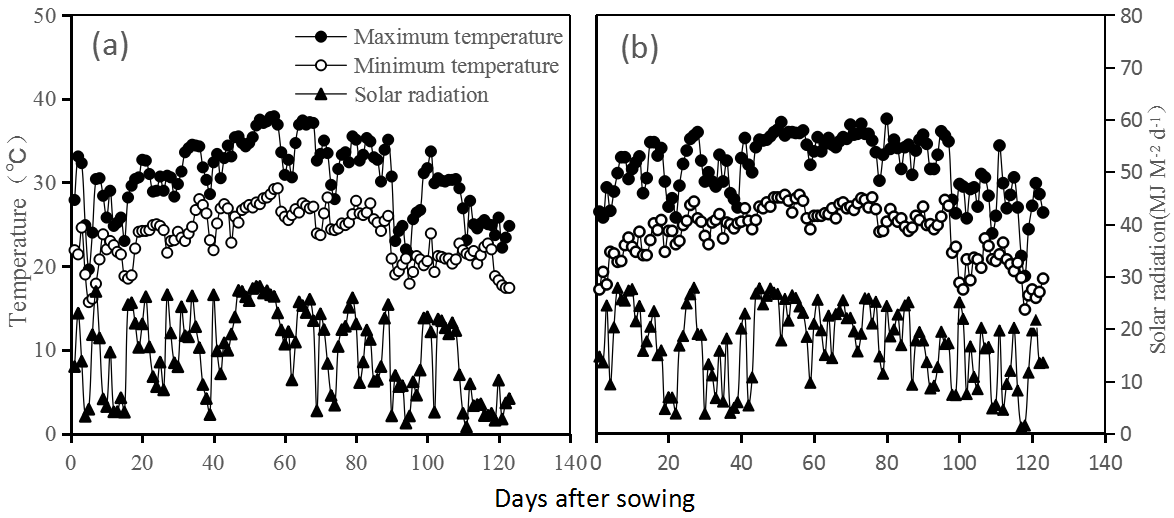

Supplement: S1 Fig — (DOCX) [file pone.0238362.s001.docx]
